# Cladribine Use in Xanthoma Disseminatum: A Rare Case Presentation and Brief Updated Literature Review

**DOI:** 10.7759/cureus.62168

**Published:** 2024-06-11

**Authors:** Matthew M Gayed, Olivia S Jew, Rami N Al-Rohil, Meenal Kheterpal

**Affiliations:** 1 Dermatology, Duke University Health System, Durham, USA; 2 Pathology and Dermatology, Duke University Health System, Durham, USA

**Keywords:** therapeutics, oncology, adverse effects, cladribine, non-langerhans cell histiocytosis

## Abstract

Xanthoma disseminatum (XD) is a rare, non-Langerhans cell histiocytosis. While treatment is notoriously difficult, 2-chlorodeoxyadenosine (cladribine) has recently emerged as a potential effective therapeutic option. Here, we describe the case of a 65-year-old male with XD who experienced significant cutaneous improvement after cladribine treatment. We also provide an updated literature review on cladribine use in patients with XD in light of reported adverse effects (AEs). While the efficacy of cladribine in XD is clear, no consensus exists for treatment duration and AE management. Hence, we strongly encourage interdisciplinary discourse involving dermatology and oncology in these cases.

## Introduction

Xanthoma disseminatum (XD) is a rare, non-Langerhans cell histiocytosis characteristically presenting as a symmetric eruption of red-brown to yellow cutaneous papules that coalesce into larger plaques [1,2]. Mucosal involvement and concomitant diabetes insipidus (DI) are common; respiratory and central nervous system involvement has also been reported [1-6]. Histopathologically, XD presents with diffuse histiocytic infiltration of the dermis with Touton giant cells [2,7,8]. Immunohistochemically, cells stain positively for CD68 and factor XIII(a) and negatively for S-100 and CD1a [2,7,8]. Treatment options for XD are limited, and the poor efficacy of several therapeutic options (systemic steroids, cyclophosphamide, fibrates, chlorambucil, azathioprine, etc.) has previously been reported [3,4]. Over the last decade, 2-chlorodeoxyadenosine (cladribine) has emerged as a promising therapeutic option [3-5]. Here, we present a notable case of XD in a patient who achieved significant response but whose treatment course was complicated by cytopenias and provide an updated review of the literature on the use of cladribine in XD. In doing so, we hope to emphasize the utilities and limitations of cladribine use for patients with XD while also emphasizing the need for interdisciplinary dialogue between dermatology and oncology for disease management. 

## Case presentation

A 65-year-old male with a history of hypertension, hypercholesterolemia, and obstructive sleep apnea presented to dermatology with a worsening rash. Approximately 15 years earlier, he reported developing red-brown papules on his right arm, which over time coalesced into larger plaques involving his torso, contralateral arm, axilla, neck, and face. He was previously treated with methotrexate, hydroxychloroquine, and topical corticosteroids without symptom resolution. 

Prior to presentation, he underwent biopsies and imaging studies with local dermatologists. Magnetic resonance imaging of the brain and orbits was significant for mild bilateral optic nerve atrophy. Punch biopsy of the right upper arm showed a nodular collection of amphophilic cells with the presence of foam cells and Touton giant cells. Immunohistochemistry revealed positive staining for CD68 and CD163 and was negative for CD1a, CD30, and S-100. Together, the pathologic and immunohistochemistry findings suggested a non-Langerhans cell histiocytosis. Collected DNA from the formalin-fixed paraffin-embedded tissue underwent BRAF mutational testing by multiplex PCR. It showed no evidence for any of the BRAF V600 mutations. 

**Figure 1:**
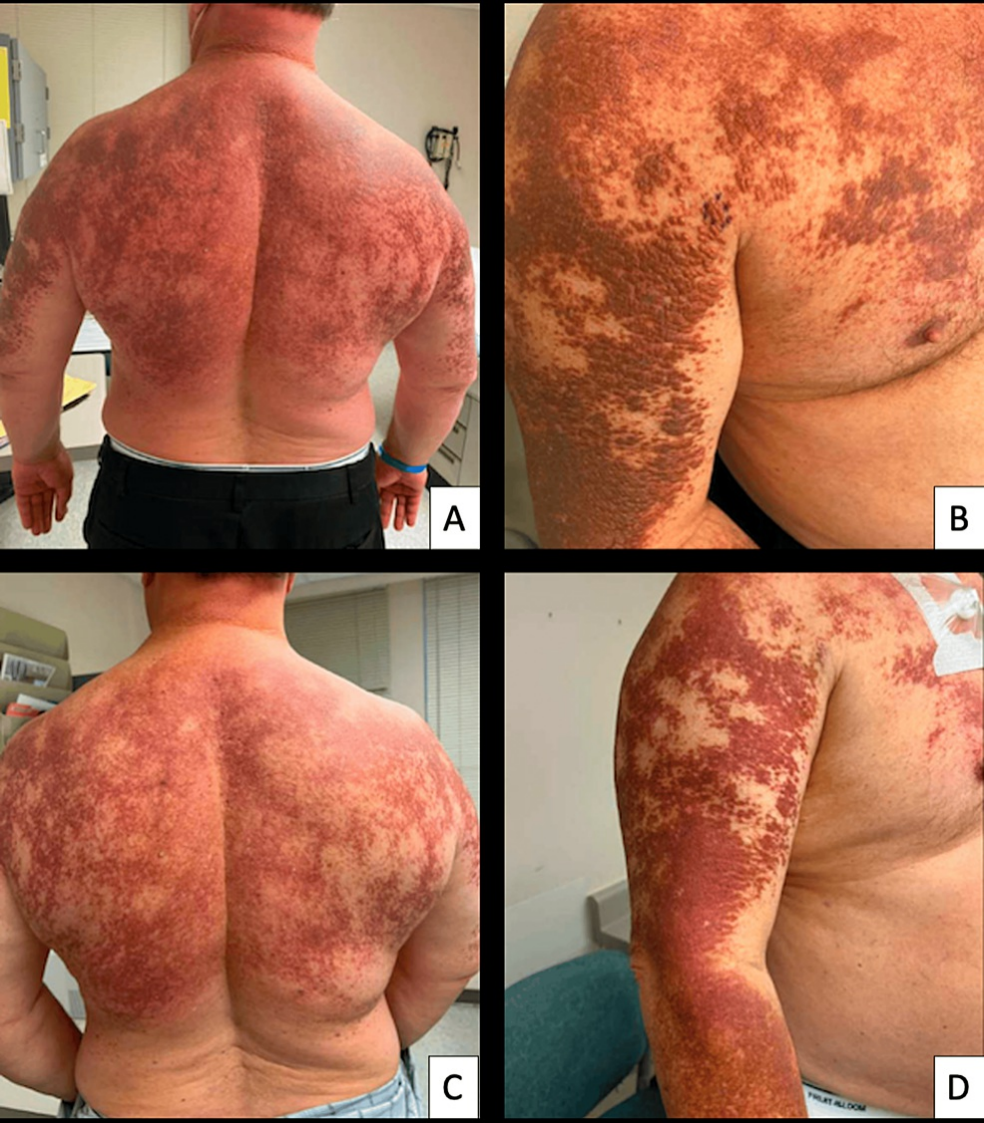
Clinical images of xanthoma disseminatum before and after cladribine Physical exam revealed reddish-brown papular lesions coalescing into plaques prior to the initiation of cladribine on the back (A) and right upper extremity (B). After six cycles of cladribine therapy, lesions on the back (C) and right upper extremity (D) exhibited flattening, reduced induration, and reduced erythema.

On presentation, physical exam was notable for numerous reddish-brown to yellow papules coalescing into plaques symmetrically distributed on the upper arms, forearms, neck, chest, abdomen, and back (Figure 1A, 1B). Numerous scattered papules were also noted on the bilateral thighs, nose, cheeks, and periorbital skin. The initial differential diagnosis included XD as well as Erdheim-Chester disease due to the cutaneous findings and bilateral optic nerve atrophy. 

**Figure 2:**
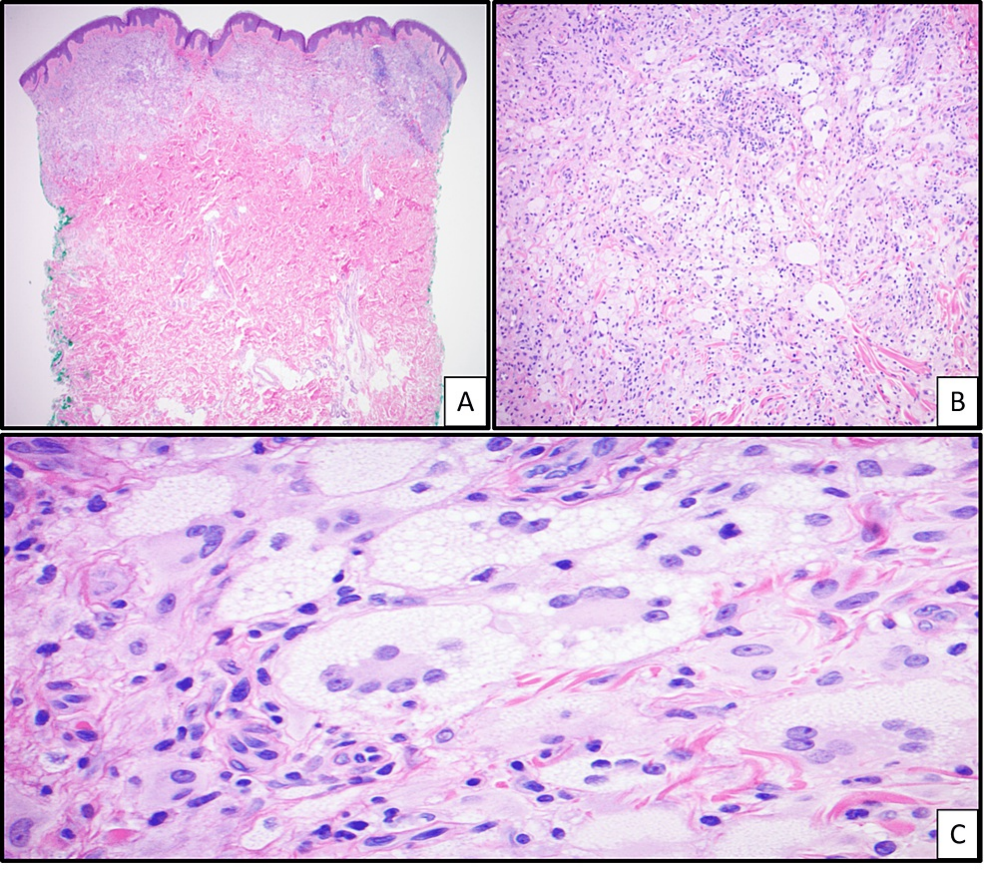
Histological images of xanthoma disseminatum Hematoxylin and eosin of skin punch biopsy reveals papillary and mid-dermal infiltrate (A, 4×). The infiltrate is composed of xanthomatous cells admixed with some lymphocytes with background dermal fibrosis (B, 10×). Among the xanthomatous cells, numerous Touton giant cells were observed (C, 40×).

Repeat punch biopsies of the right and left upper arm were performed and showed superficial and mid-dermal histiocytic infiltrate with numerous xanthomatous forms and Touton giant cells in the papillary and superficial reticular dermis, as well as mild dermal fibrosis (Figure 2). He was also noted to have borderline elevated serum osmolality (297 mOsm/kg) with normal urine osmolality and monoclonal gammopathy with M-spike (0.25 g/dL) for which he was followed by oncology; no further work-up was felt necessary at the time. Following clinicopathologic correlation, he was diagnosed with XD. He then underwent treatment with cladribine infusions at a dose of 0.14 mg/kg/day for five days/month for six cycles. 

His treatment was complicated by cytopenias. Following cycle 1, he became leukopenic (white blood cell (WBC) count of 1.3×109 cells/L) and neutropenic (absolute neutrophil count of 800 cells/mm^3^). This trend continued, and he was treated with a prophylactic course of levofloxacin during cycles 1-3. Approximately one month following cycle 6, he experienced prolonged pancytopenia and was admitted with a hemoglobin of 5.2 g/dL. He received multiple packed red blood cell infusions and was discharged after seven days. Improving hemoglobin, platelet counts, and WBC counts were noted on follow-up after discharge.

On re-evaluation in the clinic, he had complete resolution of lesions on the neck, face, and thighs. The remaining lesions on his torso and axillae had completely flattened, with residual hyperpigmented macules and patches in the involved areas (Figure 1C, 1D). Laboratory evaluation revealed stable hyperosmolality (295 mOsm/kg) and M-spike (0.32 g/dL). Hemoglobin level improved (11 g/dL) and WBC count had normalized to 5.1×109 cells/L. Given his clinical improvement, port removal and yearly follow-up with oncology and dermatology were recommended. 

## Discussion

Over the last 15 years, cladribine has emerged as a promising therapeutic option for the management of XD. Cladribine is an adenosine deaminase-resistant purine nucleoside analog thought to induce cell death by triggering deoxyribonucleic acid breaks after phosphorylation by deoxycytidine kinase [9,10]. Its toxicity in monocytes, the precursor to histiocytes, has been well established, as is its use in other histiocytic disorders [9,10].

Thus far, cladribine use for XD has been described in 23 patients across 10 case reports and three case series [3-6,8,11-18]. On review, patients were largely treated with 3-10 cycles of cladribine at a standard dose of 0.14 mg/kg/day for five days per month, with the exception of one pediatric patient [3] dosed according to body surface area. All but one patient [6] experienced marked cutaneous and mucosal response, with some experiencing complete, long-standing remission up to eight years post-completion [4]. Though 10 of the 23 patients were reported to have concomitant DI [3-5,11,13], only one patient was reported to have improvement after treatment [11]. Adverse effects (AEs) of cladribine were reported in seven patients (30%) [3,4,8,12,13,17]. Of these, five patients experienced significant cytopenias: one patient experienced asymptomatic transient leukopenia [13], one experienced grade 3 afebrile neutropenia not requiring treatment [17], one experienced grade 4 afebrile neutropenia requiring myeloid growth factors [17], one required dose reduction following six cycles due to worsening thrombocytopenia [3], and one required treatment discontinuation following three cycles due to severe thrombocytopenia [12]. Recent grey literature describing the treatment of six patients with XD on cladribine additionally supports the regular occurrence of adverse events [19].

Evidently, cladribine has been highly effective in the management of cutaneous, mucosal, and pulmonary manifestations of XD in pediatric and adult patients and shows promise in the management of lesions involving the urologic and central nervous systems. While cladribine-associated adverse events in patients with XD have been reported, management of treatment sequelae and treatment duration have not adequately been discussed. While our patient was able to complete the recommended treatment course, he required prophylactic levofloxacin during treatment and was hospitalized following cycle 6 due to significant pancytopenia. The most appropriate dosing of cladribine for the treatment of XD remains unclear. We strongly encourage interdisciplinary discourse involving dermatology and oncology when considering the use of cladribine for the treatment of XD. Such a dialogue will undoubtedly improve patient safety and disease management by encouraging routine follow-up to mitigate potential AEs as well as continual assessment of treatment benefits.

## Conclusions

Here, we describe the clinical course of a patient with XD effectively treated with cladribine but who experienced significant cytopenias as a result of treatment. Though cladribine has proven to be an effective treatment for XD in recent case reports, AEs in this patient population have thus far been underreported, and an appropriate dosing regimen remains unclear. Given this, dermatologists should work in conjunction with oncologists to coordinate the use of cladribine in patients with XD. Future research should be aimed at better understanding the role of cladribine in the treatment of XD as well as elucidating proper dosing parameters through randomized controlled trials to promote disease regression while mitigating risk.
